# Brain Tumor Biobank Development for Precision Medicine: Role of the Neurosurgeon

**DOI:** 10.3389/fonc.2021.662260

**Published:** 2021-04-26

**Authors:** Emilie Darrigues, Benjamin W. Elberson, Annick De Loose, Madison P. Lee, Ebonye Green, Ashley M. Benton, Ladye G. Sink, Hayden Scott, Murat Gokden, John D. Day, Analiz Rodriguez

**Affiliations:** ^1^ Winthrop P. Rockefeller Cancer Institute, University of Arkansas for Medical Sciences, Little Rock, AR, United States; ^2^ Department of Neurosurgery, University of Arkansas for Medical Sciences, Little Rock, AR, United States; ^3^ Division of Neuropathology, Department of Pathology, University of Arkansas for Medical Sciences, Little Rock, AR, United States

**Keywords:** biobank, brain tumor, precision medicine, precision oncology, neurosurgery

## Abstract

Neuro-oncology biobanks are critical for the implementation of a precision medicine program. In this perspective, we review our first year experience of a brain tumor biobank with integrated next generation sequencing. From our experience, we describe the critical role of the neurosurgeon in diagnosis, research, and precision medicine efforts. In the first year of implementation of the biobank, 117 patients (Female: 62; Male: 55) had 125 brain tumor surgeries. 75% of patients had tumors biobanked, and 16% were of minority race/ethnicity. Tumors biobanked were as follows: diffuse gliomas (45%), brain metastases (29%), meningioma (21%), and other (5%). Among biobanked patients, 100% also had next generation sequencing. Eleven patients qualified for targeted therapy based on identification of actionable gene mutations. One patient with a hereditary cancer predisposition syndrome was also identified. An iterative quality improvement process was implemented to streamline the workflow between the operating room, pathology, and the research laboratory. Dedicated tumor bank personnel in the department of neurosurgery greatly improved standard operating procedure. Intraoperative selection and processing of tumor tissue by the neurosurgeon was integral to increasing success with cell culture assays. Currently, our institutional protocol integrates standard histopathological diagnosis, next generation sequencing, and functional assays on surgical specimens to develop precision medicine protocols for our patients. This perspective reviews the critical role of neurosurgeons in brain tumor biobank implementation and success as well as future directions for enhancing precision medicine efforts.

## Introduction

Biobank implementation requires significant infrastructure and institutional resources. Brain tumor biobanking has been essential for advancements in diagnosis, understanding mechanisms of pathogenesis, and the development of patient derived models (1–4). Patient derived preclinical models that can assess therapies have transformed precision medicine platforms in oncology (5–7). The quality, preservation, and processing of the surgical tissue can have downstream effects on clinical translational efforts making the role of the surgical oncologist multifaceted.

Precision oncology utilizes molecular profiles to determine potential therapeutic targets (8, 9). Next generation sequencing (NGS) has allowed for the routine integration of molecular markers such as identification of oncogenic mutations. For certain brain tumors such as glioblastoma, molecular markers are routinely used for diagnosis and prediction of therapeutic response (10). We implemented a prospective adult brain tumor bank coupled with NGS to provide data and tissue samples for precision medicine efforts. Brain metastases remain the most common brain tumor in adults, and the most common malignant and non-malignant primary brain tumors are glioblastoma and meningioma, respectively (11, 12). All adult patients who were candidates for tumor resection at our tertiary referral center qualified for potential biobanking in order to build a large comprehensive compendium comprised of all tumor subtypes. In this perspective, we review our personal experience as well as describe the integral role of the neurosurgeon in providing adequate tissue samples for translational research opportunities. The goal of our efforts is to provide precision medicine options for our neurosurgical oncology patients.

## Implementation

Our tumor bank protocol was implemented in January 2019. Our paradigm was to have the neurosurgeon discuss biobanking with the patient at the time of preoperative counseling in both the outpatient and inpatient settings. Every patient with a brain tumor qualified as we did not limit enrollment by tumor subtype. Based on neurological symptoms, some patients lacked capacity and could not consent for themselves. In certain states, only a legal power of attorney can provide research consent, and a family member is not adequate. Consenting criteria for one’s institution and state can be reviewed thoroughly with the internal review board (3). During our first year of implementation, 15% of patients undergoing surgery for a brain tumor were not consented for biobanking. The top three reasons for this include workflow issues (14/29), altered mental status without adequate power of attorney (5/29), and small tumor size (3/29). For tumors <1 cm in diameter, the surgical team did not feel sending tissue for research was feasible without compromising diagnosis. Surgeons were told to aim for sending a volume of at least 5 mm^3^ for research. No maximum limit was set, and size was variable between tumor banked samples. If a patient had more than one tumor removed during a surgery, samples from each tumor were sent separately. Three was the highest number of tumors removed at one setting (n = 1) followed by two tumors (n = 2).

Once a patient has agreed to biobank consent, a nurse who has undergone specialized training will complete the consent process and documentation. We also asked all patients permission for next generation sequencing of the tumor with matched germ line samples from blood or saliva. In the first 6 months of our experience, our neurosurgery clinical nurses participated in this phase. However, given the time commitment as well as need for collection of biospecimen for germ line sequencing, our department eventually hired a separate staff member who would fulfill this function as well as go into the operating to streamline intraoperative needs.

**Figure 1:**
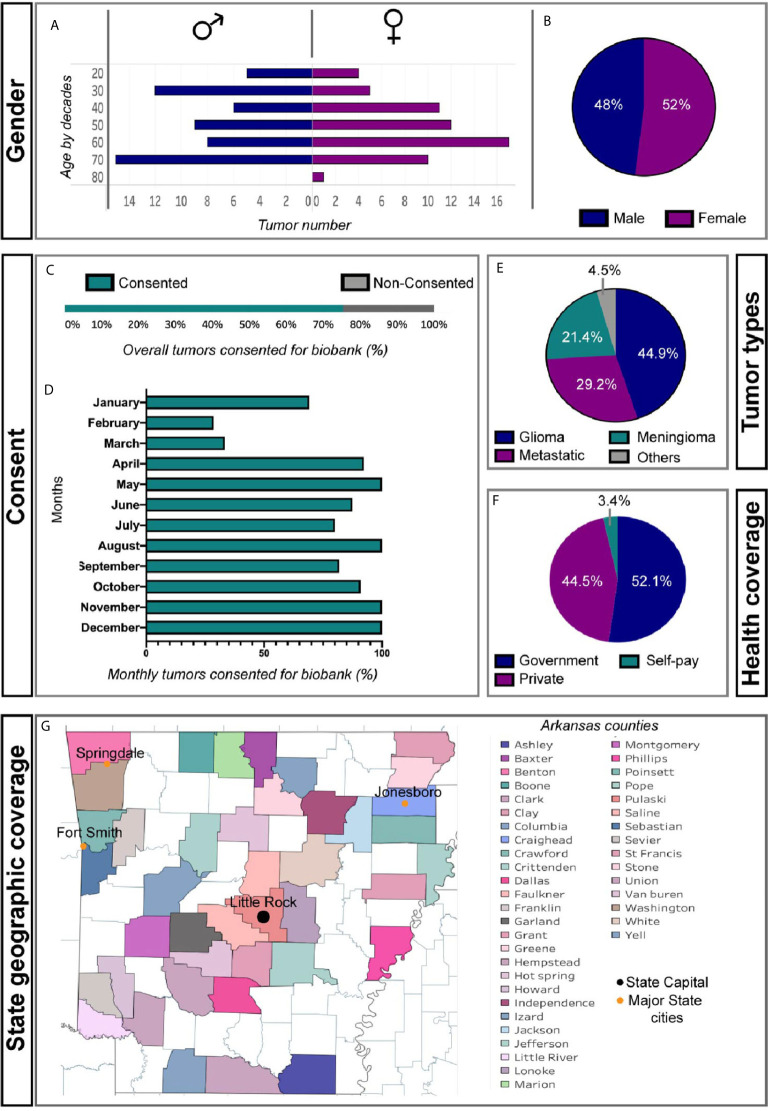
Basic patient demographics from first year of brain tumor biobank experience. **(A)** Gender and age by decade of cohort. **(B)** Percent of Male and Female patients consented **(C)** Overall and **(D)** monthly percent patients undergoing tumor resection consented for tumor biobank. **(E)** Diagnostic classification of biobanked tumors. **(F)** Insurance status of biobanked patients. **(G)** Regional distribution of patients among Arkansas counties.

In our first year the median age of patients biobanked was 59 (48%: male, 52%: female) (**Figures 1A, B**). 16% of patients were from an underrepresented minority group. Over time, the percentage of tumor patients who were consented for tumor bank enrollment was close to 100 (**Figures 1C, D**). This is most likely attributed to establishing a standard protocol, hiring dedicated tumor biobank personnel, and having quarterly departmental meetings discussing implementation and enrollment goals. The main type of tumor resected was glioma (44.9%) followed by metastases (29.2%), meningioma (21.4%), and other tumor types (4.5%) (**Figure 1E**). The majority of our patients were insured (>95%) (**Figure 1F**) and came from all regions of our state (**Figure 1G**). Five patients were from other states.

All patients who had tumor biobanking also agreed to NGS. The NGS data was utilized to identify potential actionable mutations. NGS analysis from 125 surgeries identified 11 patients had actionable mutations that could be targeted by Food and Drug Administration approved drugs. We advocate for germ line sequencing to accurately identify somatic mutations and genetic predisposition syndromes. One patient was found to have Li-Fraumeni syndrome (LFS) and underwent genetic counseling for themselves and family members. Furthermore, the knowledge of a genetic cancer predisposition syndrome such as LFS, changes the clinical follow-up as these patients often require monitoring of multiple organ systems. Patients with cancer predisposition syndrome are sent to our genetic counselor at the Cancer Institute.

NGS is performed on formalin fixed paraffin embedded pathologic tissue. The NGS sequencing we use is comprised of a targeted DNA cancer mutation panel of 595 genes and requires >50% tissue be comprised of tumor (13). Therefore, our neuropathology team determines which tissue blocks are appropriate to send for NGS profiles. Many insurance companies will not cover the cost of NGS, but we have been successful in using a company that accepts financial assistance forms making the testing free for patients that do not have insurance NGS coverage. Our NGS data is routinely integrated into both the electronic medical record and our molecular tumor board and was helpful in diagnosis. For example, in diffuse gliomas our pathologist already sends for IDH and p53 mutational status, MGMT methylation, EGFR amplification, and 1p/19q co-deletion assessment. CDK2 is a biomarker that has implications for diagnosis, prognosis and upon recurrence can identify potential therapies (14). The status of this gene is verified with our NGS panel and used by our neuropathologist and neuro-oncologist. CDK2 mutation is a potential actionable mutations and identification of actionable mutations can aid in identifying targeted treatments for both primary and metastatic brain tumors (15–18). We also obtain PD-L1 positivity level and tumor mutational burden (TMB) on all tumors which has implications for immunotherapy (IT) use. Recent data indicates that glioma tumors with low TMB are more likely to respond to IT (19). Clinical trials have demonstrated that IT can work for intracranial metastatic disease (20, 21). Clinically significant tumor mutations are also annotated within the tumor biobank data repository. These data are inserted retrospectively as NGS results typically take about 2–3 weeks to return. Our biobank tissue repository database and NGS database are linked with unique identifications numbers to maintain deidentification. Our data repository is maintained by colleagues in the department of biomedical informatics and contains patient demographics including previous treatments, pathological diagnosis information, mutations identified by NGS, and information on successful cells and/or patient models available from the sample.

## Intraoperative Considerations

**Figure 2:**
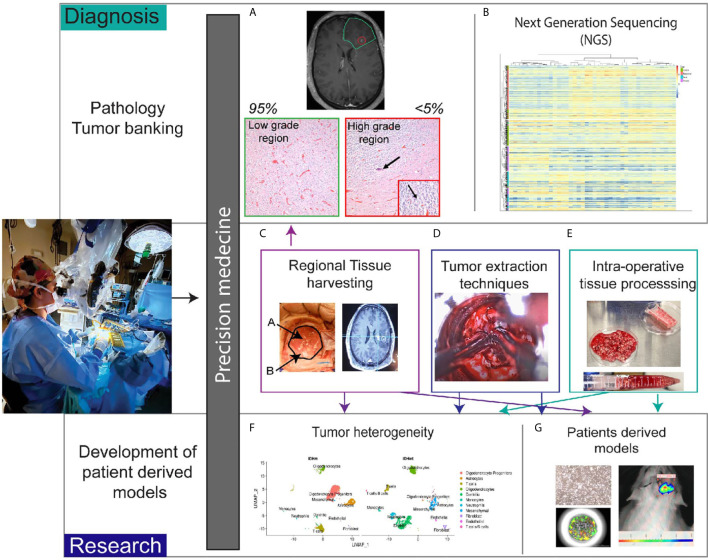
Role of the neurosurgeon in biobank for precision medicine. **(A)** Patient tumors with heterogenous regions are sent separately to pathology. Non-enhancing region corresponds with low grade histopathologic features. Enhancing region (<5% of tumor in this case) correlates with high grade histopathologic features. Arrow in the image shows calcification and the inset magnifies region of high proliferation with the arrow denoting cells undergoing cell division. **(B)** Pathologic tissue is sent for next generation sequencing. Heatmap of glioblastoma gene expression profiles stratified into classical, mesenchymal, neural and proneural subtypes. **(C)** Intraoperative tissue handling by the neurosurgeon involves regional harvesting utilizing MRI navigation **(D)** varied tumor extraction techniques such as non-thermal resection with specialized tools, and **(E)** intraoperative tissue processing with mechanical and chemical dissociation. **(F)** Once in the research laboratory, tissue can undergo single cell sequencing to determine heterogeneity. t-distributed stochastic neighbor embedding plots demonstrate differences between IDH mutant and IDH wildtype gliomas. **(G)** Tissue is also processed for patient derived models such as cell lines, organoids, and xenografts.

The neurosurgeon’s goals during operative resection include 1) obtaining tissue for accurate diagnosis and 2) resecting as much tumor as possible without causing neurological deficits. Given the known heterogeneity of many tumors such as glioblastoma, identification of diagnostic tumor regions is necessary for correct pathological grading. For example, in glioma surgery, we routinely use 5-aminolevulenic acid (5-ALA) as an adjunct to surgery. 5-ALA is converted to the fluorescent metabolite, protoporphyrin IX, by malignant glioma cells, which allows detection using a 410 nm blue light (22–25). GBM is characteristically heterogenous and can contain large areas of necrosis (26). 5-ALA is helpful to identify regions of active tumor (27). In certain glioma tumors, the area of enhancement is minimal and can comprise less than 5% of the tumor bulk. In these tumors, intraoperatively there is minimal fluorescent detection, and our institutional paradigm is to send the areas of highest 5-ALA uptake separately to the pathologist. This practice increases the likelihood of accurate pathological tumor grade since these fluorescent tumor regions often correspond to regions with high grade tumor (**Figures 2A, C**). Given our experience with fluorescence heterogeneity within the same tumor, we do send research samples from these various regions if enough tumor is available to not compromise diagnosis. In recurrent glioma cases, the patients have previously undergone chemoradiation therapy and therefore portions of the enhancing tumor may represent treatment changes. The use of 5-ALA allows for identification of viable tumor regions which are necessary to send to neuropathology for diagnosis and to provide tissue amenable for NGS processing and research.

In brain tumor biobanking, the decision of what tissue is allotted for pathology versus research applications can also be determined intraoperatively (**Figure 2**). At some centers, the tissue is sent to pathology, processed for diagnostic testing, and from there a decision is made about what portions are appropriate for research. Our paradigm differs in that we send tissue directly from the operating room (OR) to the research laboratory. Early on in the resection, we send tissue for frozen pathology and confirm a diagnosis is possible from the tissue sent. The surgeon ensures that along with the frozen pathology specimen, adequate tissue is available for diagnosis (**Figure 2A**) and in turn NGS (**Figure 2B**). If there is not enough extra viable tissue to send for research, the surgery team will notify the research team that no sample is available. However, almost always a specimen that has been selected by the operating surgeon is available for research purposes (**Figures 2C–E**). During tumor resection, we use various surgical instruments, such as the NICO myriad device, to non-thermally mechanically dissociate the tissue (28) (**Figure 2D**). This tissue is then stored in a sterile container that is on ice. Intermittently the surgeon will rinse the tubing with sterile fluid that aids in cell preservation. Cauterization is used minimally when resecting tumor tissue for research and necrotic regions are avoided. For specialized assays such as single cell sequencing, tissue viability and prevention of RNA degradation is critical for downstream experiments to be successful. Dependent on the research goals, chemical dissociation fluid can also be included to augment the mechanical dissociation (**Figure 2E**). Furthermore, multiple specimens from various regions can be sent from the OR and labeled to denote location. For example, in our glioma tumors, the periventricular region that corresponds to the subventricular zone (SVZ), the central region, and peripheral invasive region are harvested separately and annotated accordingly to notify the researchers receiving the tissue. Using neuronavigation, the regions are identified preoperatively and discussed with the research team (**Figures 2C, G**). We implemented this workflow given the extensive data supporting regional heterogeneity with disparate niches within the microenvironment (29, 30) (**Figure 2F**). These niches are exceedingly important when isolating stem cell subpopulations (31, 32).

When able, metastatic tumors are removed *en bloc* but also can be mechanically dissociated outside of the cerebrum and prior to sending for research. Mechanical dissociation in the operating room decreases processing time in the laboratory and aids in streamlining experimentation (**Figure 2E**) Dependent of the location of the brain metastasis, often a rim of the surrounding tissue within the parenchyma can be resected as well. Recent data also demonstrates heterogeneity between regions of metastatic tumors and invasion into the peritumoral border (33, 34). Therefore, even with metastatic tumor regional information is routinely annotated with the tumor tissue.

The implemented workflow we developed allows for tissue to leave the OR and arrive in the research laboratory within 1 h. The tissue remains in a sterile container on ice and is not processed by the pathology team. Decreasing the number of staff that handle the tissue reduces the chances of contamination, as most samples are used for cell culture. In some circumstances, the surgery can last hours, and we have trained our surgical team to send tissue shortly after resection at interval times rather than waiting until the completion of surgery. If a surgery extends past biobank staff work hours, we use a preservation solution for the tissue which is stored sterilely overnight at 4°C. With this protocol, we have been able to still isolate tumor cells the following work day. Our current workflow was an iterative process of continual improvement with feedback from the surgical and research teams to optimize outcomes.

## Translation Research Efforts

The focus of our biobank is to use our research towards precision medicine efforts. We hope to impact diagnosis, treatment, and ultimately improve patient survival. We review our experience to aid others in this goal. The cost of our research efforts have been supported mainly by our academic department and Cancer Institute. We hope to fully transition to extramural funding in the near future and do understand that funding can be a limitation to biobanking. At our institution, we routinely use next generation sequencing and recently have used tissue from our biobank to develop new methods for biomarker evaluation. We confirmed that third generation long read sequencing in combination with cas9 targeting can identify multiple brain tumor biomarkers in the same sample in under 48 h (35). Our research group is one of the first to utilize third generation sequencing on clinical samples and hope to expand the applications of this sequencing technology. Furthermore, sequencing costs is a barrier for biomarker assessment in many countries and therefore exploring new efficient techniques can allow for precision medicine efforts to be expanded in developing countries as well (36).

For therapeutic innovation, we acknowledge the need for accurate models of the patient’s tumor. Cell lines generated from patient samples have been critical for many discoveries in cancer biology. However, there are some disadvantages as these models do not recreate the tumor microenvironment or 3D structure of the parent tumor (37). In our first year experience, isolation of cells that could be serially passaged was possible in 43% of diffuse gliomas and 54% of brain metastases. We typically also culture isolated cells in serum free conditions to maintain stem cell populations if possible. However, it has been well established that not all tumor samples are amenable to having a cell line generated. For example, IDH mutant glioma cells are difficult to maintain in culture and typically will not maintain the IDH mutant phenotype over time (38). Currently very few brain metastases cell lines are available but researchers are working on establishing repositories for these models as well (39).

Patient derived xenografts have been the mainstay of cancer models but are expensive and have long engraftment times (40–42). Furthermore, they also are often recapitulated in an immune compromised host and with the advent of immunotherapy in cancer treatment this can be a disadvantage. For precision medicine efforts, the prolonged engraftment time can severely limit the ability to develop and test treatment options for a patient especially for cancer types (like GBM) that have a short survival. However, recent advances in 3D models, namely tumor organoids, have significantly advanced the capability of model tumors and performing therapeutic or treatment screening (37, 43–47).

In our translational neurosurgery laboratory, we have been successful in developing both 2D and 3D cell cultures from tumor specimens as well as animal models (**Figure 2G**). We hope to combine insights from our NGS data obtained during diagnosis with assays derived from patient tissue to identify therapies. This paradigm is known as functional precision medicine wherein genomics and *ex vivo* drug sensitivity screening are combined for personalized oncology therapy (48). We are currently enrolled in a prospective functional precision medicine trial for our high-grade glioma patients (NCT03561207). In this trial, surgical tissue from resection or biopsy is used to grow 3D cultures and screen a panel of drugs. During tumor extraction for this trial, the surgeon regularly communicates with the pathologist to ensure the tissue has viable tumor present. This is particularly important when sending biopsy cores in the recurrent setting. Again, the role of the neurosurgeon is critical for functional precision trials as the assay is dependent on having tissue that results in tumor cell isolation. We are currently developing an internal functional precision medicine assay for glioma and brain metastases patients.

## Future Directions

Precision medicine will eventually become routine in oncological care. Brain tumors have significant morbidity, and malignant brain tumors typically portend a grim prognosis. We foresee that a multi-omics approach will be used to predict response to therapy and identify new treatments. Advances in machine learning allow for data to be obtained from radiographic imaging (*i.e.* Radiomics) and digital pathology to enhance diagnosis and predict genomic biomarkers (49–53). These data will likely lead to the ability of predicting tumor subtype and prognosis prior to surgical resection.

Besides the identification of cancer specific tumor mutations, the advances in NGS allow for assessment of transcriptomics and epigenetics. The results of the National Cancer Institute’s Molecular Analysis for Therapy Choice (MATCH), a precision medicine trial based on identification of actionable mutations, demonstrated in a cohort of 4,687 patients that only 17.8% of patients qualified for therapy (54). In another cohort of 500 cancer patients, genomic DNA profiling was able to identify potential targets for 29.6% of patients, but increased to 43.4% with the integration of RNA sequencing and immune biomarkers (13). Tumor RNA sequencing therefore has utility in identifying potential targets when DNA based genomics does not. Vaske et al. developed a transcriptomic approach to identify significantly targets in pediatric cancers as these typically do not have actionable DNA mutations (55).

Proteogenomics (the combination of genomics with proteomics) is also beginning to be utilized to toxicity and resistance to therapies and determine precision oncology strategies. The National Cancer Institute’s Clinical Proteomic Tumor Analysis Consortium (CPTAC) has published several seminal manuscripts on various cancer types including pediatric brain cancer (56–60). We have found proteogenomics to be useful in identifying key pathways involved in metastatic progression to the brain (61). We foresee a transition to utilizing multi-omics to understand cancer landscapes and identify targetable oncogenic pathways.

Epigenetic analyses are also becoming more common to use for diagnosis and prognosis. Epigenetic alterations do not change the DNA sequence but do impact gene activity. Epigenetic changes are integral for tumor progression and in mediating chemotherapy resistance (62). The most commonly studied mechanism for epigenetics is DNA methylation, and in brain tumors this has thus far been the most well studied. MGMT methylation status is a common biomarker used to ascertain potential response to temozolomide treatment in GBM (63–65). Methylome profiles have been demonstrated to be useful in stratifying GBM patients in regards to treatment response and survival (66–68). For brain metastases, DNA methylomes have also identified unique biological features with therapeutic implications (69–71). Recently, data from methylomes of meningiomas have significantly impacted the classification and clinical management of these tumors by providing insights into prognosis and recurrence prediction (72–74). Other epigenetic alterations of importance in brain tumor pathophysiology are histone modifications. Pediatric high grade gliomas and some adults gliomas have mutations in histone 3 which impact chromatin function and gene expression (75–78). These mutations have significant implications for potential therapies and are now part of the diagnostic criteria for these tumors (79, 80). Currently, only certain epigenetic biomarkers (namely MGMT methylation status and presence of histone 3 mutations) are used for diagnosis in neuro-oncology, but as the cost of methylation sequencing decreases, we expect that epigenetic characterization will be routine and used to predict response to therapy and prognosis.

Tumor tissue sequencing is not the only source of biospecimen that can be used for precision medicine. Liquid biopsy refers to the sequencing of plasma or other biological fluid such as cerebrospinal fluid (CSF) to identify mutations (8, 81). Glioma tumor evolution can be tracked with CSF liquid biopsy and this is promising potentially differentiating tumor recurrence from treatment change (82). Methylation profiles of plasma derived liquid biopsies can be used to discriminate common primary intracranial tumors (83). These data indicate that liquid biopsies will be used clinically in the near future for brain tumor precision medicine.

## Discussion

Implementation and maintenance of a brain tumor biobank are necessary for precision medicine advancements. From our experience, we recommend the combination of NGS and biobanking as well as development of a data repository that interfaces with the electronic medical record. We have used our biobank for the development of diagnostic assays and for the development of patient derived models. Current areas of improvement include the generation of patient derived models with intact immune microenvironment components, verification of liquid biopsies as proxies for tissue analysis, and integration of multi-omics derived from sequencing as well as radiomics and digital pathology. For these improvements, neurosurgeons will play a key role and ultimately are vital team members for functional precision medicine programs.

## Data Availability Statement

The original contribution presented in the study and further inquiries can be directed to the corresponding author.

## Ethics Statement

The studies involving human participants were reviewed and approved by (IRB protocol #228443). The patients/participants provided their written informed consent to participate in this study.

## Author Contributions

Design and concept: AR. Writing of draft and data interpretation: ED, ML, BE, and AR. Review of article: all authors. All authors contributed to the article and approved the submitted version.

## Funding

ED was supported by the Translational Research Institute (TRI), grant TL1 TR003109 and UL1 TR003017 through the National Center for Advancing Translational Sciences of the National Institutes of Health (NIH). AR was supported by the Winthrop P. Rockefeller Cancer Institute Seeds of Science Grant.

## Conflict of Interest

The authors declare that the research was conducted in the absence of any commercial or financial relationships that could be construed as a potential conflict of interest.

## References

[1] Ostrom QT Devine K Fulop J Wolinsky Y Liao P Stetson L. Brain tumor biobanking in the precision medicine era: Building a high-quality resource for translational research in neuro-oncology. Neuro-Oncol Pract (2017) 4:220–8. 10.1093/nop/npw029 PMC5909804 29692920

[2] Hojat A Wei B Olson MG Mao Q Yong WH. Procurement and storage of surgical biospecimens. Methods Mol Biol (2019) 65–76. 10.1007/978-1-4939-8935-5_7 PMC6918836 30539435

[3] Harati MD Williams RR Movassaghi M Hojat A Lucey GM Yong WH. An introduction to starting a biobank. Methods Mol Biol (Humana Press Inc) (2019) 7–16. 10.1007/978-1-4939-8935-5_2 PMC6777713 30539430

[4] Im K Gui D Yong WH. An introduction to hardware, software, and other information technology needs of biomedical biobanks. Methods Mol Biol (2019) 17–29. 10.1007/978-1-4939-8935-5_3 PMC6777721 30539431

[5] Brabetz S Leary SES Gröbner SN Nakamoto MW Şeker-Cin H Girard EJ. A biobank of patient-derived pediatric brain tumor models. Nat Med (2018) 241752–61. 10.1038/s41591-018-0207-3 30349086

[6] Weeber F Ooft SN Dijkstra KK Voest EE. Tumor Organoids as a Pre-clinical Cancer Model for Drug Discovery. Cell Chem Biol (2017) 24. 10.1016/j.chembiol.2017.06.012 28757181

[7] Sachs N de Ligt J Kopper O Gogola E Bounova G Weeber F. A Living Biobank of Breast Cancer Organoids Captures Disease Heterogeneity. Cell (2018) 172:P373–86.E10. 10.1016/j.cell.2017.11.010 29224780

[8] Schwartzberg L Kim ES Liu D Schrag D. Precision Oncology: Who, How, What, When, and When Not? Am Soc Clin Oncol Educ B (2017) 37:160–9. 10.1200/edbk_174176 28561651

[9] Fernandez-Rozadilla C Simões AR Lleonart ME Carnero A Carracedo Á. Tumor Profiling at the Service of Cancer Therapy. Front Oncol (2021) 10:595613. 10.3389/fonc.2020.595613 33505911 PMC7832432

[10] Patterson J Wongsurawat T Rodriguez A. A Glioblastoma Genomics Primer for Clinicians. Med Res Arch (2020) 8:1–17. 10.18103/mra.v8i2.2034 PMC7111506 32258388

[11] Ostrom QT Wright CH Barnholtz-Sloan JS. Brain metastases: epidemiology. Handb Clin Neurol (Elsevier BV), 27–42. 10.1016/B978-0-12-811161-1.00002-5 29307358

[12] Ostrom QT Gittleman H Liao P Vecchione-Koval T Wolinsky Y Kruchko C. CBTRUS Statistical Report: Primary brain and other central nervous system tumors diagnosed in the United States in 2010–2014. Neuro Oncol (2017) 19:v1–88. 10.1093/neuonc/nox158 29117289 PMC5693142

[13] Beaubier N Bontrager M Huether R Igartua C Lau D Tell R. Integrated genomic profiling expands clinical options for patients with cancer. Nat Biotechnol (2019) 37:1351–60. 10.1038/s41587-019-0259-z 31570899

[14] Korshunov A Casalini B Chavez L Hielscher T Sill M Ryzhova M. Integrated molecular characterization of *IDH* -mutant glioblastomas. Neuropathol Appl Neurobiol (2019) 45(2):108–18. 10.1111/nan.12523 30326163

[15] Venur VA Cohen JV Brastianos PK. Targeting Molecular Pathways in Intracranial Metastatic Disease. Front Oncol (2019) 9:99. 10.3389/fonc.2019.00099 30886831 PMC6409309

[16] Han CH Brastianos PK. Genetic Characterization of Brain Metastases in the Era of Targeted Therapy. Front Oncol (2017) 7:230. 10.3389/fonc.2017.00230 28993799 PMC5622141

[17] Nørøxe DS Skjøth-Rasmussen J Brennum J Østrup O Kinalis S Nielsen FC. GENE-50. GENOMIC PROFILING AND PRECISION MEDICINE IN GLIOBLASTOMA - A PROSPECTIVE STUDY. Neuro Oncol (2017) 19:vi103–3. 10.1093/neuonc/nox168.422

[18] Young JS Prados MD Butowski N. Using genomics to guide treatment for glioblastoma. Pharmacogenomics (2018) 19:1217–29. 10.2217/pgs-2018-0078 30203716

[19] Gromeier M Brown MC Zhang G Lin X Chen Y Wei Z. Very low mutation burden is a feature of inflamed recurrent glioblastomas responsive to cancer immunotherapy. Nat Commun (2021) 12:1–7. 10.1038/s41467-020-20469-6 33441554 PMC7806846

[20] Aquilanti E Brastianos PK. Immune Checkpoint Inhibitors for Brain Metastases: A Primer for Neurosurgeons. Neurosurgery (2020) 87:E281–8. 10.1093/neuros/nyaa095 PMC7426188 32302389

[21] Becco P Gallo S Poletto S Frascione MPM Crotto L Zaccagna A. Melanoma brain metastases in the era of target therapies: An overview. Cancers (Basel) (2020) 12:1–20. 10.3390/cancers12061640 PMC7352598 32575838

[22] Barone DG Lawrie TA Hart MG. Image guided surgery for the resection of brain tumours. Cochrane Database Syst Rev (2014) 1:CD009685. 10.1002/14651858.CD009685.pub2 PMC6457761 24474579

[23] Hadjipanayis CG Stummer W. 5-ALA and FDA approval for glioma surgery. J Neurooncol (2019) 141479–86. 10.1007/s11060-019-03098-y PMC6445645 30644008

[24] Zhao S Wu J Wang C Liu H Dong X Shi C. Intraoperative fluorescence-guided resection of high-grade malignant gliomas using 5-aminolevulinic acid-induced porphyrins: a systematic review and meta-analysis of prospective studies. PLoS One (2013) 8:e63682. 10.1371/journal.pone.0063682 23723993 PMC3665818

[25] Lau D Hervey-Jumper SL Chang S Molinaro AM McDermott MW Phillips JJ. A prospective Phase II clinical trial of 5-aminolevulinic acid to assess the correlation of intraoperative fluorescence intensity and degree of histologic cellularity during resection of high-grade gliomas. J Neurosurg (2015) 124(5):1–10. 10.3171/2015.5.JNS1577 26544781

[26] Hambardzumyan D Bergers G. Glioblastoma: Defining Tumor Niches. Trends Cancer (2015) 1:252–65. 10.1016/j.trecan.2015.10.009 PMC4831073 27088132

[27] Smith SJ Diksin M Chhaya S Sairam S Estevez-Cebrero MA Rahman R. The Invasive Region of Glioblastoma Defined by 5ALA Guided Surgery Has an Altered Cancer Stem Cell Marker Profile Compared to Central Tumour. Int J Mol Sci (2017) 182452. 10.3390/ijms18112452 PMC5713419 29156557

[28] Zusman E Sidorov M Ayala A Chang J Singer E Chen M. Tissues harvested using an automated surgical approach confirm molecular heterogeneity of glioblastoma and enhance specimen’s translational research value. Front Oncol (2019) 9:1119. 10.3389/fonc.2019.01119 31750239 PMC6843001

[29] Lee JH Lee JE Kahng JY Kim SH Park JS Yoon SJ. Human glioblastoma arises from subventricular zone cells with low-level driver mutations. Nature (2018) 560:243–7. 10.1038/s41586-018-0389-3 30069053

[30] Darmanis S Sloan SA Croote D Mignardi M Chernikova S Samghababi P. Single-Cell RNA-Seq Analysis of Infiltrating Neoplastic Cells at the Migrating Front of Human Glioblastoma. Cell Rep (2017) 21:1399–410. 10.1016/j.celrep.2017.10.030 PMC5810554 29091775

[31] Cheng L Huang Z Zhou W Wu Q Donnola S Liu JK. Glioblastoma stem cells generate vascular pericytes to support vessel function and tumor growth. Cell (2013) 153:139–52. 10.1016/j.cell.2013.02.021 PMC3638263 23540695

[32] Schiffer D Mellai M Annovazzi L Caldera V Piazzi A Denysenko T. Stem cell niches in Glioblastoma: A Neuropathological view. BioMed Res Int (2014) 2014. 10.1155/2014/725921 PMC4009309 24834433

[33] Berghoff AS Rajky O Winkler F Bartsch R Furtner J Hainfellner JA. Invasion patterns in brain metastases of solid cancers. Neuro Oncol (2013) 15:1664–72. 10.1093/neuonc/not112 PMC3829586 24084410

[34] Dankner M Caron M Al-Saadi T Yu W Ouellet V Ezzeddine R. Invasive growth associated with Cold-Inducible RNA-Binding Protein expression drives recurrence of surgically resected brain metastases. Neuro Oncol (2021) 1–11. 10.1093/neuonc/noab002 33433612 PMC8408858

[35] Wongsurawat T Jenjaroenpun P De Loose A Alkam D Ussery DW Nookaew I. A novel Cas9-targeted long-read assay for simultaneous detection of IDH1/2 mutations and clinically relevant MGMT methylation in fresh biopsies of diffuse glioma. Acta Neuropathol Commun (2020) 81–13. 10.1186/s40478-020-00963-0 PMC7305623 32563269

[36] Srivathsan A Baloğlu B Wang W Tan WX Bertrand D Ng AHQ. A MinION^TM^-based pipeline for fast and cost-effective DNA barcoding. Mol Ecol Resour (2018) 18:1035–49. 10.1111/1755-0998.12890 29673082

[37] Aboulkheyr Es H Montazeri L Aref AR Vosough M Baharvand H. Personalized Cancer Medicine: An Organoid Approach. Trends Biotechnol (2018) 36:358–71. 10.1016/j.tibtech.2017.12.005 29366522

[38] Xie Y Bergström T Jiang Y Johansson P Marinescu VD Lindberg N. The Human Glioblastoma Cell Culture Resource: Validated Cell Models Representing All Molecular Subtypes. EBioMedicine (2015) 21351–63. 10.1016/j.ebiom.2015.08.026 PMC4634360 26629530

[39] Valiente M Van Swearingen AED Anders CK Bairoch A Boire A Bos PD. Brain Metastasis Cell Lines Panel: A Public Resource of Organotropic Cell Lines. Cancer Res (2020) 80:4314–23. 10.1158/0008-5472.can-20-0291 PMC7572582 32641416

[40] Candolfi M Curtin JF Nichols WS Muhammad AG King GD Pluhar GE. Intracranial glioblastoma models in preclinical neuro-oncology: neuropathological characterization and tumor progression. J Neurooncol (2007) 85:133–48. 10.1007/s11060-007-9400-9 PMC2384236 17874037

[41] Xiao M Rebecca VW Herlyn M. A Melanoma Patient-Derived Xenograft Model. J Vis Exp (2019) (147):e59508. 10.3791/59508 PMC6986799 31157772

[42] Bhimani J Ball K Stebbing J. Patient-derived xenograft models—the future of personalised cancer treatment. Br J Cancer (2020) 122:601–2. 10.1038/s41416-019-0678-0 PMC7054515 31919403

[43] Hubert CG Rivera M Spangler LC Wu Q Mack SC Prager BC. A Three-Dimensional Organoid Culture System Derived from Human Glioblastomas Recapitulates the Hypoxic Gradients and Cancer Stem Cell Heterogeneity of Tumors Found In Vivo. Cancer Res (2016) 76:2465–77. 10.1158/0008-5472.CAN-15-2402 PMC4873351 26896279

[44] Jacob F Salinas RD Zhang DY Nguyen PTT Schnoll JG Wong SZH. A Patient-Derived Glioblastoma Organoid Model and Biobank Recapitulates Inter- and Intra-tumoral Heterogeneity. Cell (2019) 180(1):188–204. 10.1016/j.cell.2019.11.036 31883794 PMC7556703

[45] Brandenberg N Hoehnel S Kuttler F Homicsko K Ceroni C Ringel T. High-throughput automated organoid culture via stem-cell aggregation in microcavity arrays. Nat BioMed Eng (2020) 4:1–12. 10.1038/s41551-020-0565-2 32514094

[46] Golebiewska A Hau AC Oudin A Stieber D Yabo YA Baus V. Patient-derived organoids and orthotopic xenografts of primary and recurrent gliomas represent relevant patient avatars for precision oncology. Acta Neuropathol (2020) 140(6):919–49. 10.1007/s00401-020-02226-7 PMC7666297 33009951

[47] Darrigues E Nima ZA Griffin RJ Anderson JM Biris AS Rodriguez A. 3D cultures for modeling nanomaterial-based photothermal therapy. Nanoscale Horizons (2020) 5:400–30. 10.1039/c9nh00628a 32118219

[48] Letai A. Functional precision cancer medicine-moving beyond pure genomics. Nat Med (2017) 23:1028–35. 10.1038/nm.4389 28886003

[49] Kurc T Bakas S Ren X Bagari A Momeni A Huang Y. Segmentation and Classification in Digital Pathology for Glioma Research: Challenges and Deep Learning Approaches. Front Neurosci (2020) 14:27. 10.3389/fnins.2020.00027 32153349 PMC7046596

[50] Karami E Soliman H Ruschin M Sahgal A Myrehaug S Tseng C-L. Quantitative MRI Biomarkers of Stereotactic Radiotherapy Outcome in Brain Metastasis. Sci Rep (2019) 9:19830. 10.1038/s41598-019-56185-5 31882597 PMC6934477

[51] Zhou H Vallières M Bai HX Su C Tang H Oldridge D. MRI features predict survival and molecular markers in diffuse lower-grade gliomas. Neuro Oncol (2017) 19:862–70. 10.1093/neuonc/now256 PMC5464433 28339588

[52] Soike MH McTyre ER Shah N Puchalski RB Holmes JA Paulsson AK. Glioblastoma radiomics: can genomic and molecular characteristics correlate with imaging response patterns? Neuroradiology (2018) 60:1043–51. 10.1007/s00234-018-2060-y PMC7193682 30094640

[53] Bhatia A Birger M Veeraraghavan H Um H Tixier F Mckenney AS. MRI radiomic features are associated with survival in melanoma brain metastases treated with immune checkpoint inhibitors. Neuro Oncol (2019) 21(12):1578–86. 10.1093/neuonc/noz141 PMC7145582 31621883

[54] Flaherty KT Gray R Chen A Li S Patton D Hamilton SR. The Molecular Analysis for Therapy Choice (NCI-MATCH) Trial: Lessons for Genomic Trial Design. J Natl Cancer Inst (2020) 112:1021–9. 10.1093/jnci/djz245 PMC7566320 31922567

[55] Vaske OM Bjork I Salama SR Beale H Tayi Shah A Sanders L. Comparative Tumor RNA Sequencing Analysis for Difficult-to-Treat Pediatric and Young Adult Patients With Cancer. JAMA Netw Open (2019) 2:e1913968. 10.1001/jamanetworkopen.2019.13968 31651965 PMC6822083

[56] Gao Q Zhu H Dong L Shi W Chen R Song Z. Integrated Proteogenomic Characterization of HBV-Related Hepatocellular Carcinoma. Cell (2019) 179:561–77.e22. 10.1016/J.CELL.2019.08.052 31585088

[57] Huang C Chen L Savage SR Eguez RV Dou Y Li Y. Proteogenomic insights into the biology and treatment of HPV-negative head and neck squamous cell carcinoma. Cancer Cell (2021) 39(3):361–79. 10.1016/j.ccell.2020.12.007 PMC7946781 33417831

[58] Krug K Jaehnig EJ Satpathy S Blumenberg L Karpova A Anurag M. Proteogenomic Landscape of Breast Cancer Tumorigenesis and Targeted Therapy. Cell (2020) 183:1436–56.e31. 10.1016/j.cell.2020.10.036 33212010 PMC8077737

[59] Hu Y Pan J Shah P Ao M Thomas SN Liu Y. Integrated Proteomic and Glycoproteomic Characterization of Human High-Grade Serous Ovarian Carcinoma. Cell Rep (2020) 33p108276. 10.1016/j.celrep.2020.108276 PMC7970828 33086064

[60] Petralia F Tignor N Reva B Koptyra M Chowdhury S Rykunov D. Integrated Proteogenomic Characterization across Major Histological Types of Pediatric Brain Cancer. Cell (2020) 183:1962–85.e31. 10.1016/j.cell.2020.10.044 33242424 PMC8143193

[61] Taylor EM Byrum SD Edmondson JL Wardell CP Griffin BG Shalin SC. Proteogenomic analysis of melanoma brain metastases from distinct anatomical sites identifies pathways of metastatic progression. Acta Neuropathol Commun (2020) 8:157. 10.1186/s40478-020-01029-x 32891176 PMC7487560

[62] Strauss J Figg WD. Using epigenetic therapy to overcome chemotherapy resistance. Anticancer Res (2016) 36:1–4. 26722021 PMC6388403

[63] Okada M Miyake K Tamiya T. Glioblastoma Treatment in the Elderly. Neurol Med Chir (Tokyo) (2017) 57:667–76. 10.2176/nmc.ra.2017-0009 PMC5735230 29081442

[64] Storey K Leder K Hawkins-Daarud A Swanson K Ahmed AU Rockne RC. Glioblastoma Recurrence and the Role of O 6-Methylguanine-DNA Methyltransferase Promoter Methylation. JCO Clin Cancer Inform (2019) 3:1–12. 10.1200/CCI.18.00062 PMC6732255 30758983

[65] Lee SY. Temozolomide resistance in glioblastoma multiforme. Genes Dis (2016) 3:198–210. 10.1016/J.GENDIS.2016.04.007 30258889 PMC6150109

[66] Fukunaga T Fujita Y Kishima H Yamashita T. Methylation dependent down-regulation of G0S2 leads to suppression of invasion and improved prognosis of IDH1-mutant glioma. PLoS One (2018) 13:e0206552. 10.1371/journal.pone.0206552 30388142 PMC6214530

[67] Wenger A Vega SF Kling T Bontell TO Jakola AS Carén H. Intratumor DNA methylation heterogeneity in glioblastoma: Implications for DNA methylation-based classification. Neuro Oncol (2019) 21:616–27. 10.1093/neuonc/noz011 PMC6502500 30668814

[68] Klughammer J Kiesel B Roetzer T Fortelny N Nemc A Nenning K-H. The DNA methylation landscape of glioblastoma disease progression shows extensive heterogeneity in time and space. Nat Med (2018) 24:1611–24. 10.1038/s41591-018-0156-x PMC6181207 30150718

[69] Orozco JIJ Knijnenburg TA Manughian-Peter AO Salomon MP Barkhoudarian G Jalas JR. Epigenetic profiling for the molecular classification of metastatic brain tumors. Nat Commun (2018) 9:4627. 10.1038/s41467-018-06715-y 30401823 PMC6219520

[70] Orozco JI Manughian-Peter AO Salomon MP Marzese DM. Epigenetic Classifiers for Precision Diagnosis of Brain Tumors. Epigenet Insights (2019) 12:2516865719840284. 10.1177/2516865719840284 30968063 PMC6444760

[71] Salomon MP Orozco JIJ Wilmott JS Hothi P Manughian-Peter AO Cobbs CS. Brain metastasis DNA methylomes, a novel resource for the identification of biological and clinical features. Sci Data (2018) 5:180245. 10.1038/sdata.2018.245 30398472 PMC6219670

[72] Cordova C Kurz SC. Advances in Molecular Classification and Therapeutic Opportunities in Meningiomas. Curr Oncol Rep (2020) 221–10. 10.1007/s11912-020-00937-4 32617743

[73] Shen L Lin D Cheng L Tu S Wu H Xu W. Is DNA Methylation a Ray of Sunshine in Predicting Meningioma Prognosis? Front Oncol (2020) 10:1323. 10.3389/fonc.2020.01323 33014773 PMC7498674

[74] Nassiri F Mamatjan Y Suppiah S Badhiwala JH Mansouri S Karimi S. DNA methylation profiling to predict recurrence risk in meningioma: Development and validation of a nomogram to optimize clinical management. Neuro Oncol (2019) 21:901–10. 10.1093/neuonc/noz061 PMC6620635 31158293

[75] Diaz AK Baker SJ. The genetic signatures of pediatric high-grade glioma: No longer a one-act play. Semin Radiat Oncol (2014) 24:240–7. 10.1016/j.semradonc.2014.06.003 PMC4170681 25219808

[76] Lowe BR Maxham LA Hamey JJ Wilkins MR Partridge JF. Histone H3 mutations: An updated view of their role in chromatin deregulation and cancer. Cancers (Basel) (2019) 11:1–24. 10.3390/cancers11050660 PMC6562757 31086012

[77] Huang T Garcia R Qi J Lulla R Horbinski C Behdad A. Detection of histone H3 K27M mutation and post-translational modifications in pediatric diffuse midline glioma via tissue immunohistochemistry informs diagnosis and clinical outcomes. Oncotarget (2018) 9:37112–24. 10.18632/oncotarget.26430 PMC6324678 30647848

[78] Wu G Broniscer A McEachron TA Lu C Paugh BS Becksfort J. Somatic histone H3 alterations in pediatric diffuse intrinsic pontine gliomas and non-brainstem glioblastomas. Nat Genet (2012) 44:251–3. 10.1038/ng.1102 PMC3288377 22286216

[79] Harutyunyan AS Krug B Chen H Papillon-Cavanagh S Zeinieh M De Jay N. H3K27M induces defective chromatin spread of PRC2-mediated repressive H3K27me2/me3 and is essential for glioma tumorigenesis. Nat Commun (2019) 101–13. 10.1038/s41467-019-09140-x PMC6425035 30890717

[80] Williams MJ Singleton WGB Lowis SP Malik K Kurian KM. Therapeutic targeting of histone modifications in adult and pediatric high-grade glioma. Front Oncol (2017) 7:45. 10.3389/fonc.2017.00045 28401060 PMC5368219

[81] Lee H Park C Na W Park KH Shin S. Precision cell-free DNA extraction for liquid biopsy by integrated micro fl uidics. NPJ Precis Oncol (2020) 4:1–10. 10.1038/s41698-019-0107-0 32133418 PMC7039987

[82] Miller AM Shah RH Pentsova EI Pourmaleki M Briggs S Distefano N. Tracking tumour evolution in glioma through liquid biopsies of cerebrospinal fluid. Nature (2019) 565:654–8. 10.1038/s41586-019-0882-3 PMC6457907 30675060

[83] Nassiri F Chakravarthy A Feng S Shen SY Nejad R Zuccato JA. Detection and discrimination of intracranial tumors using plasma cell-free DNA methylomes. Nat Med (2020) 26:1044–7. 10.1038/s41591-020-0932-2 PMC8500275 32572265

